# Atlas of Skin Diseases

**Published:** 1878-05

**Authors:** 


### BIBLIOGRAPHICAL.

Atlas of Skin Diseases. By Louis A. Duhring, M.D., Professor of Skin Diseases in the Hospital of the Univerity of Pennsylvania, etc. Philadelphia: J. B. Lippincott & Co. Price, $2.50 per part.

The third quarterly issue of this valuable production has been received, and we cheerfully commend it to the medical profession as the most valuable work of the kind with which we have met.

As of the others, this part consists of four chromo-lithographic plates of royal quarto size, with descriptive and explanatory texts on sheets of the same size, and in large and clear type. The four plates of this issue represent Eazema (Squamosum), Syphiloderma (Erythematosum), Purpura (Simplex) and Syphiloderma (Papulosum et Pustulosum.)

